# *PSEN1* Compound Heterozygous Mutations Associated with Cerebral Amyloid Angiopathy and Cognitive Decline Phenotype

**DOI:** 10.3390/ijms22083870

**Published:** 2021-04-08

**Authors:** Ilaria Palmieri, Marialuisa Valente, Lisa Maria Farina, Simone Gana, Brigida Minafra, Roberta Zangaglia, Orietta Pansarasa, Daisy Sproviero, Alfredo Costa, Claudio Pacchetti, Anna Pichiecchio, Stella Gagliardi, Cristina Cereda

**Affiliations:** 1 Genomic and Post-Genomic Unit, IRCCS Mondino Foundation, 27100 Pavia, Italy; ilaria.palmieri@mondino.it (I.P.); orietta.pansarasa@mondino.it (O.P.); daisy.sproviero@mondino.it (D.S.); cristina.cereda@mondino.it (C.C.); 2 Department of Molecular Medicine, University of Pavia, 27100 Pavia, Italy; 3 Laboratory of Clinical Pathology Microbiology and Genetics, SS. Annunziata Hospital, 74100 Taranto, Italy; marialuisa.valente@asl.taranto.it; 4 Advanced Imaging and Radiomics Center, Neuroradiology Department, IRCCS Mondino Foundation, 27100 Pavia, Italy; lisa.farina@mondino.it (L.M.F.); anna.pichiecchio@mondino.it (A.P.); 5 Medical Genetics Unit, IRCCS Mondino Foundation, 27100 Pavia, Italy; simone.gana@mondino.it; 6 Parkinson and Movement Disorders Unit, IRCCS Mondino Foundation, 27100 Pavia, Italy; brigida.minafra@mondino.it (B.M.); roberta.zangaglia@mondino.it (R.Z.); claudio.pacchetti@mondino.it (C.P.); 7 Unit of Behavioral Neurology, IRCCS Mondino Foundation, 27100 Pavia, Italy; alfredo.costa@mondino.it; 8 Department of Brain and Behavioral Sciences, University of Pavia, 27100 Pavia, Italy

**Keywords:** cerebral amyloid angiopathy, CAA, *PSEN1*, next-generation sequencing, NGS, compound heterozygous mutations, stop-gain variant, splice variant

## Abstract

Cerebral amyloid angiopathy (CAA) is a cerebrovascular disorder caused by the deposition of amyloid beta-peptide (Aβ) aggregates. Aβ aggregates lead to vessel rupture and intracerebral hemorrhages, detected by magnetic resonance imaging (MRI). Presenile CAA is usually genetically determined by mutations in the amyloid precursor protein (*APP*) gene. However, mutations after codon 200 in the presenilin 1 (*PSEN1*) gene have been reported to facilitate CAA onset. Here, we analyzed the genetic bases in a patient of 55 years old affected by CAA and cognitive decline. DNA was isolated and genetic analysis was performed by Next-Generation Sequencing (NGS). RNA was extracted and retro-transcribed to perform segregation analysis by TOPO-TA cloning. WB analysis was carried out to check the impact of the mutations on protein. Two compound heterozygous mutations in *PSEN1* exon 10, such as a novel stop-gain mutation (c.1070C > G) and a pathogenic splice variant (c.1129A > T), were found by NGS. Both mutations altered the presenilin 1 protein, truncating its C-terminal portion. This is the first case of CAA and cognitive decline caused by two compound mutations in *PSEN1*. With this report, we suggest extending the genetic analysis to *PSEN1* when cerebral microbleeds are observed by MRI investigation in a patient affected by presenile cognitive decline.

## 1. Introduction

Cerebral amyloid angiopathy (CAA; OMIM: 605714) is a type of cerebrovascular disorder defined by the deposition of amyloid-beta (Aβ) peptides in meningeal and cortical vessels [1]. The Aβ deposition leads to fragile vessels prone to rupture, causing intracerebral hemorrhages (ICH) mainly located in the cortex, subcortex, and leptomeninges [2]. Magnetic resonance imaging (MRI) is suited for the identification of both small or chronic cortical hemorrhages, and non-hemorrhagic (presumed ischemic) sequelae of this disease, which can also be correlated with cognitive impairment and slower gait speed in CAA patients [3,4]. The incidence of CAA increases with age, although sporadic CAA rarely appears in individuals younger than 65 and less often in individuals in their 50s. Indeed, cases of “presenile” CAA are usually genetically determined, mainly by mutations in the amyloid precursor protein (*APP*) gene; however, mutations in the presenilin 1 (encoded by *PSEN1* gene), especially occurring after codon 200, have been reported to facilitate CAA onset [5]. Interestingly, the same genes are involved in AD, which shares the same Aβ deposition in the brain, and CAA occurs in up to 90% of Alzheimer’s disease (AD; OMIM: 607822) cases [6]. Here, we present the first case of a patient diagnosed with CAA and cognitive impairment caused by compound heterozygous mutations within the exon 10 of *PSEN1*, including a never-described nonsense mutation with pathogenic effects on the protein function.

## 2. Results

### 2.1. Clinical Findings

A 54-year-old man with 11 years of education was admitted to the Department of Neurology of the IRCCS Mondino Foundation due to gait ataxia associated with cognitive decline. Motor symptoms onset was at 52 years old: gait ataxia with fall in anteropulsion and, at the same time, the patient referred cognitive decline, forgetfulness of meetings or details of stories, and he kept on repeating the same questions. These symptoms gradually worsened, driving, money management, hobbies, and social relations were not maintained as before. In addition, as the disease progressed, mild dysarthria and difficulties finding the right words occurred, followed by emotional lability with frequent and brief episodes of apathy, sadness, or irritability with verbal outbursts.

The patient had a four-year history of hypertension well-controlled with regular use of ramipril. There were no exposures to toxic substances or drugs. Family history was unremarkable for cognitive disorders. General physical exam was normal, at neurological examination gait ataxia, parkinsonism, and pyramidal signs were observed. The speech was mildly dysarthric. Neuropsychological assessment was performed: recent episodic memory and visuospatial perception were the most affected functions, while memories from the distant past were only partially impaired.

**Figure 1:**
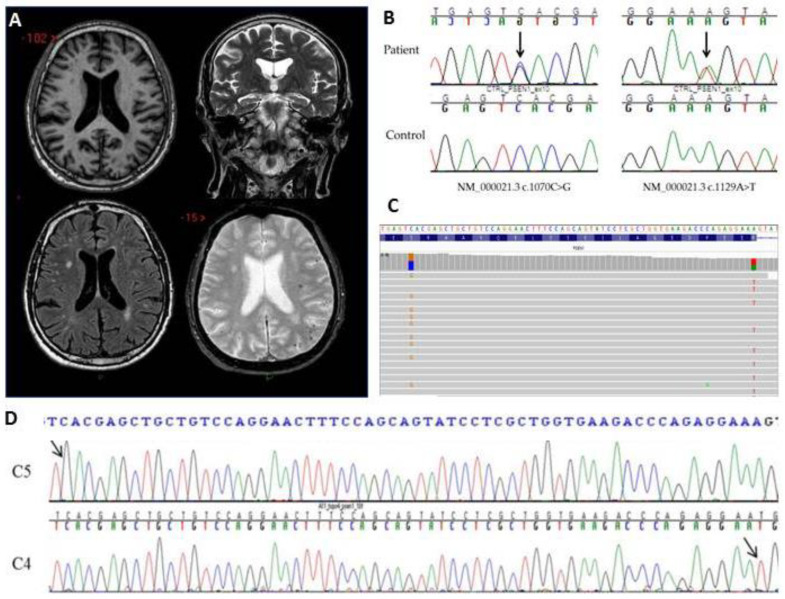
(**A**) Brain MRI: Axial T1 weighted image (upper left) shows a slight enlargement of the fronto-parietal sulci and of the posterior sylvian fissures; Coronal T2-weighted image (upper right) shows initial diffuse brain atrophy (temporal horns width with Schelten’s Medial temporal lobe atrophy (MTA) score scale scored as 2 [10]; Axial FLAIR (lower left) shows multiple multifocal asymmetric white matter lesions, mainly involving the deep and subcortical bi-hemispheric white matter; Axial T2-GE image (lower right) shows punctate multifocal hypointensity located hemorrhages, located mainly in the cortico-subcortical junction in the left hemisphere; (**B**) Electropherogram showing the heterozygous variant c.1070C > G in the exon 10 of *PSEN1* in the patient compared to a control sequence and the electropherogram showing the heterozygous variant c.1129A > T in the exon 10 of *PSEN1* in the patient compared to a control sequence; (**C**) IGV visual inspection of the two variants. Reads that read the c.1070C > G variant (orange) do not read the c.1129A > T variant (red) and vice versa; (**D**) Electropherograms from two different clones: the clone C5 carries only the c.1070C > G (arrow) variant while the clone C4 carries only the c.1129A > G variant (arrow).

Electroencephalogram (EEG), and fluorodeoxyglucose-positron emission tomography (FDG-PET) were unremarkable. The patient underwent MRI examination by Gyroscan Philips 1.5 T equipment with T1 and T2 weighted sequences for clinical routine. The MRI showed multifocal subcortical microhemorrhages mainly at the left posterior hemispheric site, suggesting a differential diagnosis with brain amyloid angiopathy (Figure 1A). Since the patient was lost to follow up, it was not possible to perform the lumbar puncture and the amyloid-PET and to confirm the progression to AD. Thus, he was diagnosed as multiple domain amnestic mild cognitive impairment complicated by movement disorders in probable CAA based on clinical and neuropsychological features and specific neuroimaging findings.

### 2.2. Genetic Analysis and In Silico Prediction

Through NGS analysis, we identified two variants in exon 10 of the *PSEN1* gene: a novel c.1070C > G nucleotide variation that results in the nonsense p.Ser357Ter mutation and the already reported c.1129A > T variant, which falls in the last nucleotide of the exon that results in the p.Arg377Trp amino acid change (Figure 1B) [7,8,9]. Both variants were absent either in our laboratory internal cohort and in all the international population databases taken into consideration. Multiple lines of computational evidence supported a probably deleterious effect on the gene product by either the stop-gain or the splice mutations (PolyPhen-2 = 1.0; SIFT = 0). All living family members declined genetic testing.

### 2.3. Variants Segregation

Since the two variants were only 59 nucleotides apart, NGS read mates were able to cover both genomic locations. IGV visual inspection of the NGS read pairs suggests that the two mutations were on two different alleles (Figure 1C). The data was then confirmed using the TOPO-TA cloning experiment since all living family members declined genetic testing. As expected, we found transfected colonies carrying the amplicon with only the c.1070C > G variant and colonies carrying the amplicon with only the c.1129A > T nucleotide change (Figure 1D).

### 2.4. Alteration of Splicing Analysis

**Figure 2:**
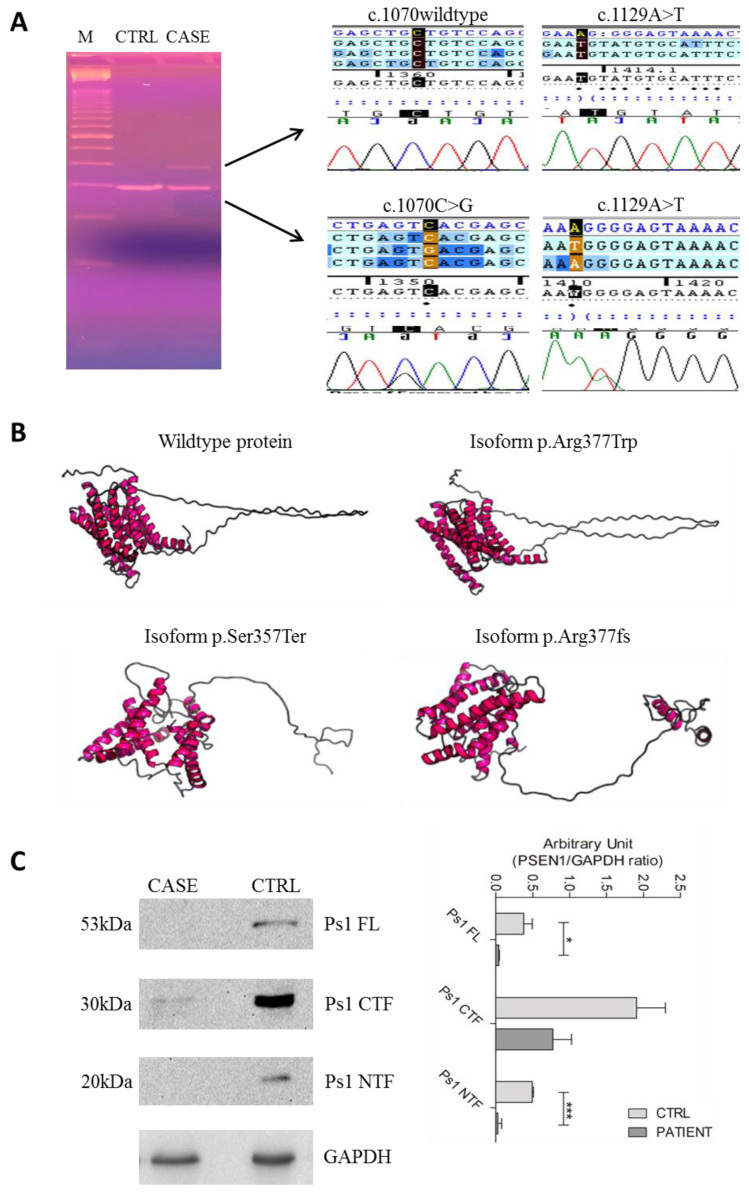
(**A**) Gel electrophoresis showing the bands corresponding to the amplified cDNA of the patient (CASE) and of the control (CTRL) and electropherograms from the cDNA sequencing: the upper band is wildtype at the c.1070 site and shows the c.1129A > T variant and an aberrant sequence corresponding to the intronic sequence; the lower band shows both variants; (**B**) 3D structure of the wildtype presenilin 1 (upper left), of presenilin 1 with the p.Arg377Trp mutation (upper right), of presenilin 1 with the p.Ser357Ter mutation (lower left); of presenilin 1 with the p.Arg377fs mutation (lower right); (**C**) WB and densitometric analysis of the presenilin 1 in the patient and in a healthy control (CTRL). M: marker; kDa: kilodalton; FL: full length; NTF: N-terminal fragment; CTF: C-terminal fragment. * *p*-value < 0.05; *** *p*-value < 0.001.

Since the c.1129A > T variant falls in the last nucleotide of exon 10 of PSEN1, we evaluated possible deleterious effects on splicing. HSF3 revealed alterations in the donor splice site AAAgtatgt of PSEN1, leading to a weakening of the site (−15.3% MaxEnt). This variant broke also another donor (GAGGAAAgt) and acceptor (CCCAGAGGAAAgta) splice sites (respectively −44.4% and −12.32% HSF Matrices). This data suggests that the c.1129A > T has deleterious effects on splicing. To investigate possible splicing alterations, cDNA has been obtained from isolated RNA, and the region comprising exons 9–11 has been amplified to look at the allele sizes. Through gel electrophoresis we found two different PCR products: one at the expected size (250 nucleotides) and the other one of around 50 nucleotides higher (Figure 2A). Amplicon sequencing of the upper band revealed the c.1129A > T variant in the homozygous state, followed by the retention of 43 intronic nucleotides, while the sequencing of the lower band showed both variants in the heterozygous state. This data suggests that the aberrant splicing did not occur 100% of the time, leading to the formation of a small percentage of protein containing the p.Arg377Trp amino acidic change.

### 2.5. D Protein Structure

Through Raptor X, the wild-type protein (Figure 2B), the p.Arg377Trp isoform, the p.Arg377fs splice isoform, and the p.Ser357Ter isoform were compared. p.Arg377Trp leads to a slight alteration of the protein 3D structure; the p.Arg377fs leads to a frameshift and to the introduction of a stop codon after 32 amino acids, giving rise to a protein with an aberrant and shorter C-terminal portion; the p.Ser357Ter leads to a conformational change in the protein structure with the formation of an even shorter C-terminal portion. WB analysis and the relative densitometry confirmed the almost absence of the full-length presenilin 1 protein as well as the C-terminal portion in the patient compared to the control. Only the N-terminal portion of the protein was present in both case and control. However, the overall levels of presenilin 1 result lower in the patient (Figure 2C).

## 3. Discussion

Herein, we identified for the first time two mutations in a compound heterozygous state within *PSEN1* in a patient affected by CAA and cognitive decline: a novel stop-gain variant (c.1070C > G; p.Ser357Ter) and an already reported mutation (c.1129A > T; p.Arg377Trp). The p.Arg377trp variant was previously described in a French patient affected by early-onset AD and in an Italian case affected by an atypical late-onset form of AD with epilepsy and frontotemporal atrophy, however, no functional studies were done to elucidate the pathogenicity of this mutation [7,8]. Only recently, the p.Arg377Trp has been reported in another Italian patient affected by late-onset AD, linking the cortical amyloid deposition to this variant [10]. However, this mutation has never been associated with the CAA phenotype. For the first time, we investigated the effect on splicing of the c.1129A > T nucleotide change and the effect on the presenilin 1 structure. The c.1129A > T variant falls in the last nucleotide of exon 10, leading to aberrant splicing that generates either a full-length wild-type transcript, and a transcript retaining 43 nucleotides of the following intron, leading to a frameshift and to the formation of an altered protein. The frameshift causes the incorporation of an anticipated stop-codon after 32 amino acids resulting in a protein of 408 instead of 467 amino acids. The amino acidic incorporation occurs in the unstructured loop of presenilin-1 that links the transmembrane (TM) domains VI to the TM-VII, eventually altering the C-terminal amino acid sequence. Additionally, the described patient carried also the novel p.Ser357Ter variant which gives rise to a protein lacking the last three TM domains. Given the implication of presenilin-1 in the proteolytic cleavage of APP and the correlation between PSEN1 mutations beyond the codon 200 and CAA onset, we suggest that the two mutations found in our patients are in charge of his clinical phenotype [5]. In conclusion, we have reported for the first time a patient affected by CAA and cognitive decline caused by two compound mutations in *PSEN1*, whose severe phenotype relies on the summative effect of the splice-site mutation and of the novel stop-gain mutation. With this study, we suggest extending the genetic analysis also to *PSEN1* when cerebral microbleeds suggestive for CAA, thus hemorrhages restricted to lobar, cortical, or cortical-subcortical regions, are observed at MRI investigation in a patient affected by presenile cognitive decline.

## 4. Materials and Methods

### 4.1. Genetic and In Silico Analyses

Genomic DNA from peripheral blood was extracted following standard procedures after obtaining the informed consent from the patient and the approval of the ethics committee of the IRCCS Mondino Foundation. NGS was performed using the Focused Exome capture library (Agilent Technologies, Santa Clara, CA, USA), following the SureSelectQXT Target Enrichment for Illumina Multiplexed Sequencing protocol. Briefly, paired-end libraries were prepared, pooled, and loaded into a MiniSeq System (Illumina, San Diego, CA, USA). FastQ file generation and bioinformatic analysis were performed as described in Filosto et al. [11]. Produced VCF were processed with eVAI software (https://evai.engenome.com/, enGenome, Pavia, Italy; accessed on 19 December 2017) for annotation and VarSome webtool (https://varsome.com/; accessed on 20 January 2020) was used for variant classification according to ACMG criteria [12,13].

Pathogenic mutations of interest were validated by direct Sanger sequencing (primers upon request) and aligned to the reference genomic sequence (NG_007386.2) using Sequencher 4.8 software. Variants frequency was checked in the major population databases such as GnomAD (Genome Aggregation Database; http://gnomad.broadinstitute.org/; accessed on 08 January 2018), 1000 Genomes (http://1000genomes.org/; accessed on 08 January 2018), and ESP (Exome Sequencing Project; http://evs.gs.washington.edu/EVS/; accessed on 08 January 2018).

Pathogenicity was assessed using in silico prediction software: PolyPhen-2 (http://genetics.bwh.harvard.edu/pph2/; accessed on 08 January 2018) and SIFT (Sorting Intolerant From Tolerant; http://sift.bii.a-star.edu.sg/; accessed on 08 January 2018). HSF3 (Human Splicing Finder 3; http://umd.be/Redirect.html; accessed on 22 November 2018) was interrogated for splice site alterations prediction. Lastly, a visual inspection of NGS-aligned reads with the Integrative Genomic Viewer tool (IGV) was done to verify the “cis/trans” localization of the variants of interest [14].

### 4.2. Allele Sequencing

Peripheral blood mononuclear cells (PBMCs) were isolated from blood using Histopaque^®^-1077 (Sigma-Aldrich, St. Louis, MO, USA) following manufacturer’s instructions, and RNA from was extracted isolated by Trizol reagent (TRIzol, Sigma-Aldrich, St. Louis, MO, USA) according to the manufacturer’s instructions. 1 µg of the extracted RNA was subsequently retrotranscribed into cDNA with the iScript cDNA Synthesis Kit (Bio-Rad, Hercules, CA, USA) and *PSEN1* exon10 amplification was carried out using the MyTaq^®^ DNA Polymerase (Bioline, Memphis, TN, USA). cDNA amplicons were checked on a 3% Agarose gel. Amplicons were extracted from the gel using the NucleoSpin Gel and PCR clean-up (Macherey-Nagel, Düren, Germany) kit, following manufacturer’s instructions. Purified bands were then sequenced using the 3130xl Genetic Analyzer (Applied Biosystems, Foster City, CA, USA). As for the Sanger validation, sequences were aligned to the reference coding sequence (NM_000021.4) using the Sequencher 4.8 software.

### 4.3. TOPO^®^TA Cloning

The nucleotide sequence of the exon 10 of the *PSEN1* gene was amplified from the genomic DNA and the PCR product was cloned into the plasmid pCR4-TOPO by T-A ligation (Invitrogen, Carlsbad, CA, USA) according to the manufacturer’s instructions. Chemically competent E. Coli DH5α™-T1R were then transformed and plated on LB-Agar plates containing 50 μg/mL ampicillin and 50 μg/mL kanamycin to get colonies. A total of 10 colonies (C1–C10) were picked and cultivated in 5 mL LB-Broth containing antibiotics. Plasmid DNA was then extracted from each colony with NucleoSpin^®^ Plasmid (Macherey-Nagel, Düren, Germany) and directly Sanger sequenced.

### 4.4. Protein Characterization

Raptor X (http://raptorx.uchicago.edu/; accessed on 22 November 2018) was used for in silico analysis of the protein structure. Immunoblot was done to assess Presenilin-1 content and size. Soluble protein extract was obtained using the RIPA buffer, from PBMCs of the patient and of healthy control. The protein content was quantified and 30 μg of the soluble extract was loaded onto a 12.5% SDS–PAGE gel (Bio-Rad, Hercules, CA, USA). After electrophoresis, samples were blotted onto a polyvinylidene fluoride (PVDF) membrane (Trans-blot, Bio-Rad, Hercules, CA, USA) subsequently incubated with the anti-Presenilin-1 antibody (GTX116016, GeneTex, Irvine, CA, USA; dilution 1:1000) overnight at 4 °C. Immunoreactivity was detected using donkey anti-rabbit (NA9340, GE Healthcare, Boston, MA, USA; dilution 1:8000) and bands were visualized using ECL Select (Ge Healthcare). Rabbit polyclonal anti-GAPDH (GTX100118, GeneTex, Irvine, CA, USA; dilution 1:1000) was used as a total soluble loading control. Densitometric analysis of the bands was performed using the ImageJ software (version number 1.51, http://rsb.info.nih.gov/ij/; accessed on 12 August 2020) and GraphPad Prism v7.

## Data Availability

The genetic data presented in this study are available on request from the corresponding author.

## Abbreviations

Aβ	amyloid-beta
AD	Alzheimer’s disease
APP	amyloid precursor protein
CAA	cerebral amyloid angiopathy
EEG	electroencephalogram
FDG-PET	fluorodeoxyglucose-positron emission tomography
ICH	intracerebral hemorrhages
MRI	magnetic resonance imaging
MTA	medial temporal lobe atrophy
NGS	next-generation sequencing
PBMCs	peripheral blood mononuclear cells
*PSEN1*	presenilin 1
TM	transmembrane
